# Monitoring Genomic Structural Rearrangements Resulting from Gene Editing

**DOI:** 10.3390/jpm14010110

**Published:** 2024-01-19

**Authors:** Susan M. Bailey, Erin M. Cross, Lauren Kinner-Bibeau, Henry C. Sebesta, Joel S. Bedford, Christopher J. Tompkins

**Affiliations:** 1 Department of Environmental and Radiological Health Sciences, Colorado State University, Fort Collins, CO 80523, USA; joel.bedford@colostate.edu; 2 KromaTiD, Inc., Longmont, CO 80501, USA; erin.cross@kromatid.com (E.M.C.); lauren.kinner@artisancells.com (L.K.-B.); hsebesta@tensentric.com (H.C.S.)

**Keywords:** directional genomic hybridization, gene editing, DNA repair, structural variants, chromosome aberrations

## Abstract

The cytogenomics-based methodology of directional genomic hybridization (dGH) enables the detection and quantification of a more comprehensive spectrum of genomic structural variants than any other approach currently available, and importantly, does so on a single-cell basis. Thus, dGH is well-suited for testing and/or validating new advancements in CRISPR-Cas9 gene editing systems. In addition to aberrations detected by traditional cytogenetic approaches, the strand specificity of dGH facilitates detection of otherwise cryptic intra-chromosomal rearrangements, specifically small inversions. As such, dGH represents a powerful, high-resolution approach for the quantitative monitoring of potentially detrimental genomic structural rearrangements resulting from exposure to agents that induce DNA double-strand breaks (DSBs), including restriction endonucleases and ionizing radiations. For intentional genome editing strategies, it is critical that any undesired effects of DSBs induced either by the editing system itself or by mis-repair with other endogenous DSBs are recognized and minimized. In this paper, we discuss the application of dGH for assessing gene editing-associated structural variants and the potential heterogeneity of such rearrangements among cells within an edited population, highlighting its relevance to personalized medicine strategies.

## 1. Introduction

### Structural Variants Arise from the Mis-Repair of DNA Double-Strand Breaks

Genome editing, or genetic engineering, particularly when employing an autologous treatment strategy, represents the apex of personalized medicine. A patient’s own cells are harvested and DNA edited to repair a specific genetic error or target a specific disease, and then returned to the patient as a truly personalized therapy. Fundamental to genome editing systems, such as clustered regularly interspaced short palindromic repeats (CRISPR) and the CRISPR-associated protein (Cas9), is the requisite induction of targeted DNA double-strand breaks (DSBs) to specific base sequences, and subsequent inactivation or replacement of the targeted or closely associated sequences [1,2,3,4]. However, because no genome editing technology or DNA repair pathway is 100% error-free, low-frequency but potentially genotoxic, structural variants are often observed in parallel with the desired edit(s) [5].

Organisms elegantly handle the endogenous production and repair of DSBs in order to accomplish essential biological processes. Concurrently, they must also deal with the potentially adverse consequences of mis-repair of DSBs, regardless of whether such breaks occur naturally or are produced from exogenous sources, such as restriction endonucleases, chemical agents, and ionizing radiations. The DNA damage response involving the repair and rejoining of DSBs in mammalian systems is accomplished via two main pathways: (1) non-homologous end joining (NHEJ), which directly ligates broken ends together and so can be error-prone, and (2) homologous recombination (HR) or homology directed repair (HDR), which relies on a template and so is relatively more precise. Canonical or classic NHEJ (c-NHEJ) is the primary pathway for repair of DSBs throughout the cell cycle, and when lacking, alternative NHEJ (alt-NHEJ) can rejoin broken DNA ends using microhomology [6,7]. By taking advantage of these critical cellular repair pathways, DNA targets can be inactivated or corrected via contemporary gene editing strategies. Targeted DSBs induced by guided endonucleases have an increased potential for mis-repair among broken DNA ends since they can be relatively close in time and proximity to other DSBs in the cell. Just as for DSBs induced by other sources, such as ionizing radiations, mis-repair events result in the creation of structural variants, including inversions, deletions, translocations, and even more complex chromosome aberrations [5,8,9,10]. A simple rearrangement, such as an inversion, requires the mis-repair of two concurrent DSBs, while complex rearrangements involve three or more DSBs, and so the risk of formation of both simple and complex structural variants increases in direct proportion to the number of on- and off-target edits occurring in an individual cell. In a non-clonal population of edited cells, there is typically a distribution of low-frequency structural variants arising from the mis-repair of on-target, off-target, and random endogenous DSBs.

Such aberrations have potentially large implications as they can contribute to genomic instability, carcinogenesis, and/or lead to a growth advantage for a potentially genotoxic variant giving rise to a sub-clonal population of cells [11,12,13]. DSB mis-rejoining events that produce structural variants are distinct from editing errors and classical off-target effects in which an edit is faulty or occurs at the wrong location, or where the resulting DSBs are rejoined to retain or restitute original chromosome continuity. To accurately assess induction of potentially undesirable genomic structural variants, direct measurements by single-cell analyses are clearly advantageous, as they do not rely on either pooled DNA from a heterogeneous cell population (as for sequencing methods that are not single-cell based) or the bioinformatic reconstruction of the genome.

**Figure 1:**
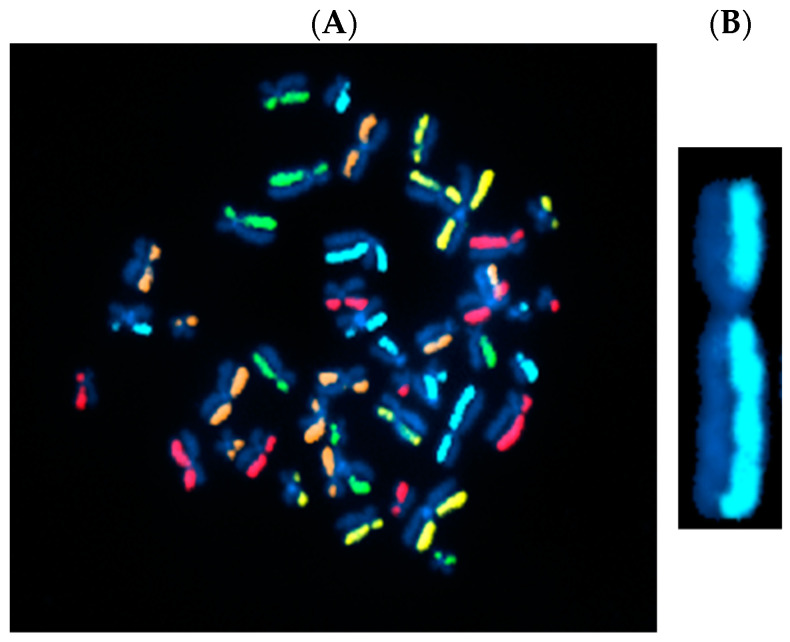
Directional genomic hybridization (dGH). (**A**) Normal, untreated human metaphase chromosome spread (peripheral blood mononuclear cells: PBMCs) illustrating strand-specific hybridization of single-stranded unique probes to the entire genome (5-color dGH SCREEN). It can be readily appreciated that, overall, there are very few structural variants in normal cells [16]. (**B**) Enlargement of a single chromosome 2 showing no structural rearrangements or inversions.

Directional genomic hybridization (dGH) is a cytogenomics-based strand-specific methodology uniquely capable of providing structural variation information on a cell-by-cell basis and at high resolution (Figure 1). One particularly important benefit of dGH over other cytogenetic methodologies is that it can reveal previously undetectable abnormalities, such as small inversions (detected at >5 Kb, significantly smaller than a G-band with a lower limit of detection of ~5–15 Mb), and sometimes referred to as “cryptic” [14,15]. Utilizing dGH, we previously showed that inversion frequencies were influenced by age and smoking status (just as for translocations) [16], and also demonstrated next-generation sequencing’s inability to detect any of the breakpoint junctions in clones containing such rearrangements, which were readily detectable by dGH [17].

Structural variants or rearrangements, such as inversions (intra-chromosomal) and translocations (inter-chromosomal), form when the broken ends of two or more nearby DSBs are incorrectly realigned and mis-rejoined, forming an exchange aberration [9,18,19]. Concurrent DSBs can arise from both endogenous and exogenous sources and, similar to exposure to ionizing radiation, include the intended gene editing process itself, off-target effects, and spontaneous and/or metabolically related DSBs, the presence of which can be detected in several ways. For example, following induction of a DSB, phosphorylation of the chromatin-associated histone variant H2AX occurs (γ-H2AX) and spreads for distances up to 1 Mb in both directions from the break site, localization of which can be visualized as discrete foci by immunocytochemistry [20,21]. The kinetics of γ-H2AX foci formation and resolution can also be monitored because they disappear as DSBs rejoin and phosphatases dephosphorylate the γ-H2AX residues. The γ-H2AX assay is well-established and widely used to detect and quantify DSB formation and disappearance in both basic research and clinical applications [21,22,23,24,25]. It is of relevance in the present context that γ-H2AX foci are copiously produced in normal, untreated cells in S-phase (detected via incorporation of BrdU during brief pulse labeling), demonstrating that DSBs are formed during the natural process of DNA replication [26].

**Figure 2:**
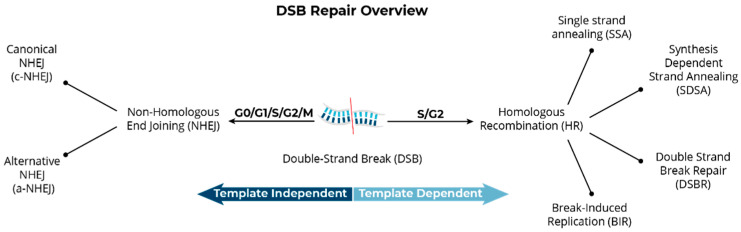
Overview of primary DSB repair pathways in mammalian cells. Pathway choice is determined by multiple variables, including cell type, stage of the cell cycle, genomic location of the DSB, and the availability of repair factors.

As previously mentioned, the primary DSB repair pathways active in mammalian cells are NHEJ (c-NHEJ and alt-NHEJ) and HR or HDR [27,28,29,30,31,32]; HR-based repair pathway choices include single-strand annealing (SSA) and breakage-induced replication (BIR) [30,31] (Figure 2). The NHEJ pathway is regarded as error-prone since it is not a high-fidelity process with respect to restoration of the original base-pair sequence around the DSB site; NHEJ invokes a cascade of proteins that resect the broken ends (deletion) and then re-joins them [32]. It is important to appreciate that the NHEJ pathway does not function for the sole purpose of repairing DSBs that occur spontaneously or as needed during DNA replication, or even to cope with the effects of exogenously encountered DSB-producing agents, like ionizing radiations. NHEJ is also fundamental to the V(D)J recombination process that enables development of remarkably diverse repertoires of T and B lymphocytes, an essential component of the immune response for detecting and coping with a wide assortment of foreign antigens [33,34]. Another major physiological mechanism of DSB formation and NHEJ-mediated repair occurs during the leptotene stage of meiosis, which engages the SPO11 endonuclease to avoid creation of complex chromosome rearrangements [35].

The other major pathway of DSB repair in mammals, HR or HDR, requires the use of a homologous DNA template to ensure higher fidelity or “error-free” DSB repair. However, HR is essentially inactive during G_1_/G_0_ phases of the cell cycle, primarily due to the absence or very low levels of RAD51 and lack of a nearby homologous repair template, i.e., a post-replication sister chromatid [36,37]. In contrast, NHEJ is active throughout all phases of the cell cycle and is predominantly responsible for the rejoining of DSBs induced by ionizing radiations or by other means, including gene editing processes [9,38,39,40,41,42]. Cells that lack functional NHEJ systems are hypersensitive to chromosomal aberration induction when exposed to gamma radiation [8,9,11,13]. The exchange of non-homologous broken DNA ends can result in mis-rejoining events even in cells with intact NHEJ when several DSBs are introduced at the same time, resulting in deletions, inversions, and translocations [9].

The current interest in evaluating mis-repair or mis-rejoining events, structural variation, genotoxicity, and chromosomal/genomic instability in genome-edited populations has intensified due to observations of these events in CRISPR-Cas9 editing systems [5,10]. Although much of the focus has been on the induction of unintended off-target DSBs and their consequences, the potential genotoxic consequences of on-target DSBs are also an important outcome that require further exploration. For example, a recent study showed that CRISPR-Cas9 editing increased the probability of chromothripsis, a catastrophic genotoxic event that causes chromosomal shattering as a result of on-target DSB formation [43,44]. Chromothripsis has been associated with a variety of potential causal mechanisms, including isolation of one or several chromosomes in a micronucleus [45], telomere crisis [46], and multiplex translocations [47,48]. Consistent with these findings, structural variants, such as translocations and deletions, have been found to persist at low levels in edited T cells months after infusion into patients [49]. These structural variants most frequently arose as a consequence of on-target DSBs and included the edit site breakpoints. Although no adverse effects have yet been linked to the population in this study, the potential oncogenic consequences of the resulting variants cannot be ignored [50,51].

Whole-genome sequencing (WGS) is widely used for evaluating induction of structural variants, but WGS approaches do not unambiguously identify such rearrangements, nor do they provide a comprehensive picture of the frequency of aberrations within a heterogenous population of cells, as occurs when batches of cells are edited using CRISPR technology. Many potentially harmful rearrangements may be present in only a fraction of cells, yet this level of heterogeneity is not discernable by sequencing methods that rely on DNA isolated from a pooled cell population. Furthermore, sequencing typically utilizes double-stranded DNA, which does not provide direct structural information on inverted sequences; therefore, detection of inversions requires bioinformatic reconstruction rather than direct identification. This approach is not simple analytically and often requires very sophisticated analysis pipelines using long-read sequencing [17,52].

The difficulties associated with some sequencing and analysis approaches for detecting structural variants, which in some cases can easily be seen in every cell of a population by cytogenetic observations, have been demonstrated [17]. One study estimated error rates in the range of 20–100% per editing-induced break, depending on the locus at which the DSB occurred and the number of Cas9-induced cuts being introduced concurrently [53]. In CRISPR-edited cells, multiple unintended head-to-tail insertions, as well as megabase scale deletions and complex structural variants were not detected using conventionally applied PCR, resulting in a high number of cells that were falsely identified as being successfully edited [54,55,56,57,58]. The above structural variants are sometimes associated with fusion genes and underlie a variety of malignancies [57]. Other pertinent studies that compare multiple platforms for the investigation of structural genome variants include [59,60,61].

Finally, although sister chromatid exchange (SCE) events are not in themselves structural variants, they are commonly used as an indicator of chromosomal instability [62]. SCE frequencies are elevated in patients with various cancers associated with genomic instability, for example in Bloom Syndrome, the quintessential SCE disorder [6]. Unlike chromosomal translocations, inversions, and ring structures that are produced via the NHEJ-mediated mis-joining of DSBs, SCEs arise during DNA replication and require HDR [63]. Normally, SCEs are non-recurrent events that appear as a random distribution within a population, whereas inversions, as true structural rearrangements, can be stable and passed on to daughter cells over many cell generations (i.e., they are transmissible and recurrent within a population). Other proxies of genomic instability, such as chromatid-type breaks and gaps, arise only as a result of an event that occurred at or after replication in the cell cycle immediately prior to the mitosis in which they are observed [18]. A high frequency of these events (relative to controls) several cycles after editing can be a measure of ongoing genomic/chromosomal instability [64]. To our knowledge, a permanent or persistent increase in the background levels of structural variants, specifically inversions, and/or replication-related instability biomarkers resulting from gene editing processes has not been widely studied. Moreover, proper controls in such studies, e.g., a non-edited population from the same source as the edited cells, is essential. Therefore, a unified approach and validated, *unbiased*, single-cell methodology capable of measuring a comprehensive spectrum of new structural variants per cell generation, as well as other hallmarks of instability over time, are needed [65].

## 2. Materials and Methods

dGH has been described in detail previously [66]. Here, for experiments involving T cells, stimulated and un-stimulated human CD8+ T cells from healthy donors were prepared according to the standard dGH protocol for T cells and harvested at several timepoints post-nucleofection with high-specificity, low-specificity, and non-targeting guideRNA and CRISPR/Cas9 ribonucleoprotein complexes; samples were then shipped to KromaTiD for analyses. For experiments involving PBMCs, blood samples were transferred into PB-Max Karyotyping medium (Gibco, Grand Island, NY, USA) and incubated at 37 °C to stimulate T-cell proliferation. After approximately 24 h, bromodeoxyuridine and bromodeoxycytidine (BrdU/BrdC) (Chem-impex, Wooddale, IL, USA), were added to cultures for incorporation during a single round of DNA replication. Cells were arrested in the first mitosis with a Colcemid (Gibco, Grand Island, NY, USA) block at 48–52 h post-stimulation, harvested, fixed in 3:1 methanol:acetic acid (Fisher Scientific, Waltham, MA, USA), and metaphase spreads prepared using standard cytogenetic techniques [66].

Slides with metaphase chromosome spreads singly substituted with BrdU/BrdC, were selectively photolyzed by UV treatment, followed by exonucleolytic degradation of the nicked DNA to remove the newly replicated strand in each metaphase chromosome. For directional genomic hybridization (dGH), single-stranded, unidirectional, tiled oligos for each target of interest were designed and hybridized to metaphase spreads, which were then counterstained with DAPI (Vectashield, Vector Laboratories, Newark, CA, USA) and imaged on an Applied Spectral Imaging Harmony system (Applied Spectral Imaging, Carlsbad, CA, USA) using a 100X objective. Assay specifications indicating expected signal patterns in the reference (diploid) genome, as well as signal patterns indicating structural variations at the loci of interest, were used to define variant analyses protocols. Images of dGH-assayed metaphase cells were analyzed for structural variations present at the loci of interest.

## 3. Results

### 3.1. Directional Genomic Hybridization (dGH) Provides a Direct and Genome-Wide Visualization of Structural Variants

**Figure 3:**
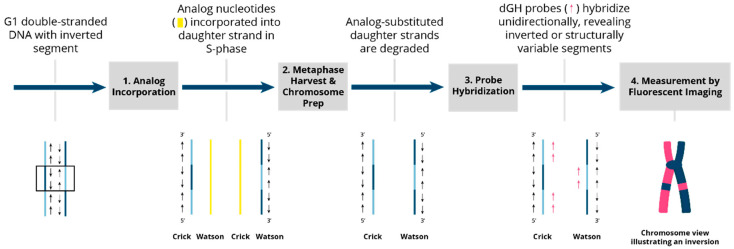
Overview of the dGH process and inversion detection. Two DSBs followed by an intra-change mis-repair (mis-rejoining) event in G1 result in an inverted segment of genomic DNA (shown within the box). **1.** Analog incorporation. The genomic DNA, including the inverted segment, is replicated in the presence of the photosensitive analog nucleotides BrdU and BrdC during a single S-phase. These analogs are incorporated into the newly synthesized DNA strands and cells are arrested in the first metaphase to ensure that the analogs are incorporated solely into newly replicated daughter strands. **2.** Metaphase harvest and chromosome preparation. Slides are exposed to UV light, which preferentially nicks the daughter strands at sites of analog incorporation and targets them for exonuclease degradation. After daughter-strand exonuclease degradation, chromosomes are left with original parental strands that are complementary to one another and of opposite 5′-to-3′ orientation (anti-parallel). **3.** Probe hybridization. dGH single-stranded probes complementary to either one or the other single-stranded chromatid are designed against the reference sequenced genome to hybridize in a directionally specific manner. **4.** Fluorescent imaging. The unique directionality of each chromatid leaves probes hybridized to a single chromatid of the chromosome, creating a fluorescently labeled “light” strand and a non-fluorescently labeled “dark” strand. Any inverted segments are readily visualized as a fluorescence pattern “switching” to the “dark” side.

**Figure 4:**
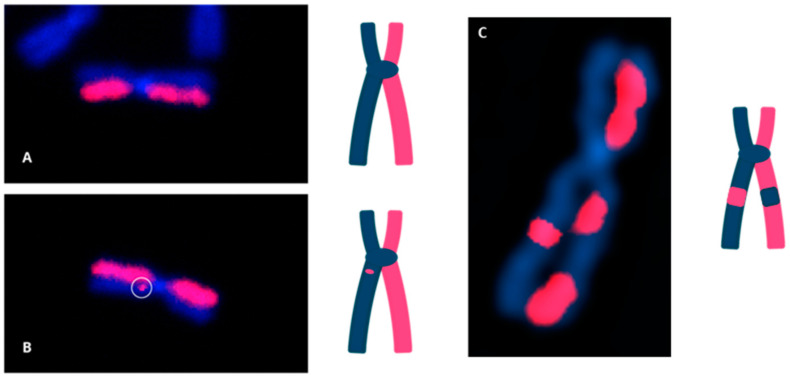
dGH detection of chromosomal inversions (structural variants). (**A**) Image of a normal chromosome 1 with no inversion. (**B**) Chromosome 2 with a single, small inversion; estimated size: ~1–5 Mb. (**C**) Chromosome 4 with a single, large inversion; estimated size: 15–45 Mb.

**Figure 5:**
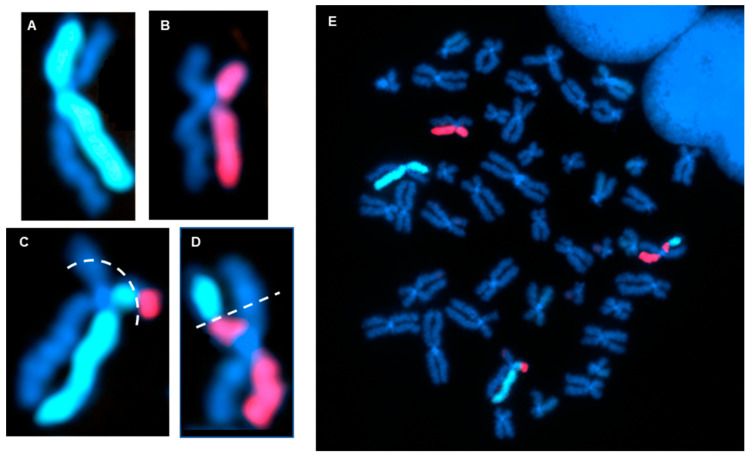
dGH detection of a reciprocal translocation. (**A**,**B**) Example of a normal dGH staining pattern for chromosome 2 (**A**) and chromosome 11 (**B**), showing no translocation or inversion events. (**C**,**D**) dGH staining pattern showing a balanced translocation between homologs of chromosomes 2 and 11. Dotted lines represent estimated breakpoints. (**E**) Full metaphase chromosome spread of the cell containing (**A**–**D**).

Directional genomic hybridization (dGH) identifies structural variants within single cells, providing information on genomic heterogeneity and the distribution of chromosomal rearrangements within edited and/or exposed cell populations [66,67]. In addition to the detection and measurement of structural variants arising from radiation exposure or induced by various methods of genome editing, dGH has been used for a variety of other applications, including oncogenic fusion gene tracking, genetic disease characterization, the detection of instability, and characterization of cancer cell lines [15,16,68,69,70]. dGH assays can also distinguish between recurrent and non-recurrent events in individual cells, e.g., to definitively identify true inversions vs. SCEs, by including telomere markers or designing specific targeted dGH assays [71]. The dGH methodology is based on the strand-specificity of Chromosome-Orientation Fluorescence Hybridization (CO-FISH) and requires (1) incorporation of BrdU/BrdC throughout a single cycle of DNA replication (S-phase), (2) collecting mitotic cells immediately following incorporation, and (3) photolytic nicking and removal of the newly synthesized, singly-substituted DNA strands [72]. This strategy renders each sister chromatid single-stranded, with the remaining parental strands being the anti-parallel complement of each other. In other words, the product is a chromosome in which the sister chromatids are single-stranded Watson/Crick (5′-to-3′ orientation) complements of each other. Directional hybridization is achieved with collections of single-stranded DNA probes whose sequences are unique to a particular chromosome and specifically designed to hybridize either to the Watson or the complementary Crick strand (i.e., all selected unique sequences have a similar orientation; they all have the same 5′-to-3′ directionality), but not both, as occurs with double-stranded DNA probes. The fluorescent tagging of the probes enables visualization and quantification of directionally specific genomic rearrangements, such as inversions, since any change in directionality results in a signal “switch” from one sister chromatid to the other [15,66]. The dGH methodology is summarized diagrammatically in Figure 3 and illustrated as applied to cells in Figure 4. Moreover, interchromosomal events, such as translocations, can be simultaneously and quantitatively measured using dGH (Figure 5); the fluorescent signal moves from chromosome to chromosome rather than from chromatid to chromatid (side to side). Note that the translocation between chromosomes 2 and 11 shown in Figure 5 would also be detected by classical whole chromosome painting, whereas inversions, such as the one shown in Figure 4, would only be visible with dGH.

### 3.2. Whole-Genome Discovery vs. Targeted Detection Using dGH

**Figure 6:**
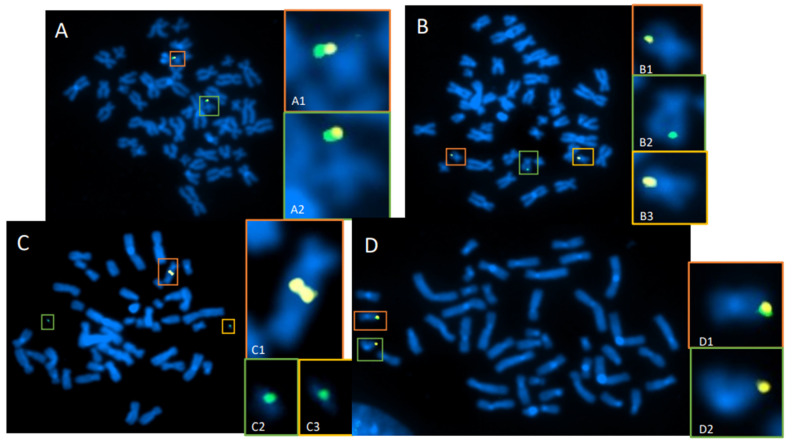
Targeted dGH detection of translocations in CRISPR/Cas9-edited T cells. T cells were transfected with CRISPR-Cas9 and guide RNAs for an edit site located on chromosome 19 (Chr19). Two probes (green and yellow) were designed to bracket the Chr19 edit site. (**A**) Cell with normal assay configuration, where yellow and green probes are co-localized on each homolog of Chr19 (**A1**,**A2**). (**B**) Normal homolog (**B1**) with normal signal pattern, and a reciprocal translocation of the green probe to an off-target chromosome (**B2**,**B3**), likely occurring at the cut site on Chr19. (**C**) Example of a cell containing an unbalanced translocation between both homologs of Chr19, resulting in a dicentric chromosome containing both yellow signals (**C1**) plus two separate acentric fragments containing the green signal (**C2**,**C3**). (**D**) Normal homolog (**D1**) with normal signal pattern, and an edit-site resection, where deletion of a large region adjacent to the edit site resulted in loss of the green signal on one Chr19 homolog (**D2**).

Whole-genome and targeted dGH assays can be used to simultaneously detect all classes of intrachromosomal and interchromosomal structural variants that occur in batches of edited cells. A summary of various dGH assay formats is provided in Table 1. For samples in which the location, type, incidence, and/or prevalence of a structural variant is unknown, whole-genome dGH can be used to track (or discover) structural deviations from the baseline of a reference genome. Within gene editing applications, dGH can be used to assess heterogenous global mis-repair events in CRISPR/Cas9-edited cell populations, where probes can be designed to target specific gene(s) of interest, including target edit sites and/or inserted transgenes, allowing for the direct assessment of specific loci in a genome (Figure 6A–D). Targeted dGH can provide information about baseline structural variation of the target site prior to editing for comparison with edited samples to determine the rate of edit-related structural variants and unintended off-site changes. Edit-site translocations (Figure 6B,C), resections (Figure 6D), and complex rearrangements, such as chromothripsis, are readily detected using this assay format. Targeted and whole-genome dGH assays can be freely multiplexed in custom formats to obtain comprehensive datasets for variants and thereby provide mechanistic insights. The data obtained with dGH assays can be used for process optimization of the nuclease, guide strand, and/or delivery strategies. Importantly, all dGH assays deliver data on a single cell or cell-by-cell basis, which can then be tabulated to demonstrate the inherent heterogeneity of structural variation in populations of edited cells.

### 3.3. Measurement of the Products of Mis-Repair in Batches of Edited Cells

In a simple gene editing context, DSBs induced at two homologous edit sites result in four free broken DNA ends that must be rejoined. In batches of edited cells, dGH detection of structural variations arising from mis-alignment of broken ends included:

− Reciprocal translocations between the edit site on non-homologous chromosomes.
− Inversions between edit sites on the target chromosome and a different site on the same chromosome, as well as translocations between edit sites on the target chromosome and various sites on different chromosomes.
− Complex variants that defied simple naming convention definitions.
− Chromothripsis products and micronuclei.

**Figure 7:**
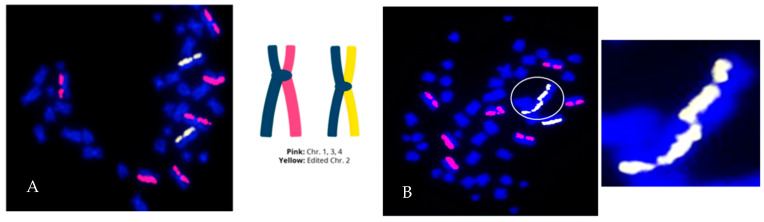
Genome editing can result in a heterogenous population of normal and abnormal cells. Human CD8+ T cells were edited with CRISPR/Cas9 ribonucleoprotein complexes. (**A**) Example of a structural error-free cell (with respect to the paint probes). The edited chromosome is painted yellow (2 homologs) and 3 putatively unedited chromosomes (2 homologs each) are painted pink. (**B**) Cell containing a complex edit-site-associated mis-repair event involving two edited homologs of chromosome 2, resulting in a dicentric chromosome (circled and enlarged), and copy number gain (Ch2). The cell is also aneuploid for chromosome 1 (trisomy).

DSBs can be introduced intentionally at edit-directed sites, randomly by endogenous metabolic and DNA replication processes, and they can occur spontaneously at fragile sites, such as minisatellites [30,73,74,75,76]. As a result, every cell in an edited population has a small but non-negligible probability of having concurrent breaks open at/near the edit site and other locations in the genome. The combination of accurate and faulty repair outcomes results in a structurally heterogeneous cell population after editing. While most of the cells may well be successfully edited and structurally normal, the successfully edited population can also be contaminated with cells harboring undesirable structural abnormalities, which can include single, multiple, and complex variants (Figure 7).

The formation of multiple break sites is associated with an increased incidence of non-reciprocal translocations [77]. When multiple edits are attempted with CRISPR/Cas9 and other editing systems, the number of DSBs increases correspondingly, thereby increasing the potential for mis-rejoining events. This includes “complex” exchange-type variants, which are possible when three or more DSBs exist concurrently [78]. In a triple knockout system, for instance, there are at least six desired concurrent DSBs per diploid G_1_ cell or twice that number in post-replication G_2_ cells, along with an indeterminant number of random breaks, all of which must be correctly rejoined to avoid the formation of structural variants. We note that evidence of instability was also observed in edited cells, as replication-related SCEs were detected at random locations throughout the genome.

Our work and that of others over many years, primarily involving ionizing radiation exposure, demonstrated that introduction of multiple DSBs increased the chances of cell death, aberrant mitosis, and/or mis-rejoining events that resulted in structural variants or chromosomal rearrangements [8,79,80,81,82,83,84]. An example of this scenario is shown in Figure 6, where a site on Chr19 is edited using CRISPR-Cas9. Even in this relatively simple system with a single target edit site, the number of translocations, edit-site resections/deletions, and other mis-repair events were elevated compared with non-edited controls. The complex cellular milieu in which gene editing occurs makes detecting and understanding the effects of mis-repair and structural variantion critical to all therapeutic editing applications [56,85,86].

## 4. Discussion

### 4.1. Classical Cytogenetics Techniques Do Not Provide a Comprehensive Assessment of Potential Structural Outcomes of Gene Editing

Classical molecular cytogenetic FISH techniques rely on the fluorescent probe-based capture of chromosome-specific single or low-copy-number sequences (Table 2). None of these approaches are capable of whole-genome detection of all potential structural variants. Inversions, in particular, have been notoriously difficult to detect with traditional cytogenetics approaches [87]. However, taking advantage of the reverse orientation of an inverted region within a chromosome (namely, utilizing the strand-specificity and directionality of dGH), high-resolution detection of previously cryptic aberrations is now possible. Moreover, all structural abnormalities, such as translocations, detected by classical methods are simultaneously revealed by dGH [15].

### 4.2. Assessment of Genomic Structural Variation Using dGH Complements Bioinformatic Sequencing Techniques Used to Predict and Measure Classical Off-Target Effects Associated with Gene Editing

Current approaches to sequencing-based structural variant detection are listed in Table 3. The identification of structural variants relies on errors in alignment, which generate a high rate of false positives, especially in highly repetitive regions. Although sequencing analysis algorithms for enhanced detection of variants are available, no single algorithm currently exists that can precisely identify all structural variants, particularly inversions [9,89].

Although sequencing-based approaches can unquestionably be useful for the analysis and identification of classical off-target edits, they are not well suited for addressing cellular heterogeneity within a population or for the detection of rare structural variants. Valuable single-cell information is lost and the frequencies of specific variants, like inversions, cannot be adequately analyzed. Moreover, rare signals become diluted and undetectable. Even single-cell sequencing does not adequately address cellular heterogeneity problems since it is not feasible in practice to analyze sufficiently large numbers of single cells to assess the spectrum of abnormalities in any real population of edited cells.

Sequencing technologies that combine several of the techniques outlined in Table 3 with novel methodologies are available for screening of classical off-target edits. Such combination strategies can resolve some of the issues associated with each approach individually. Examples include GUIDE-Seq, Circle-Seq^TM^, Change-Seq^TM^, and UDiTaS^TM^, among others [90,91]. These methods are ideal for the detection of mis-edits, including off-target edits and editing errors. However, when used alone, these methods still do not provide cell-by-cell information. The reliance on bioinformatic calculation of structure can also confound their ability to detect and differentiate between structural variant types. For instance, the patterns induced by tandem duplications and novel insertions are difficult to distinguish from one another [92]. Importantly, a number of emerging and relevant studies compare multiple platforms for investigation of structural variants that highlight the associated limitations [59,60,61].

### 4.3. Indirect Detection of Structural Variants through Fusion Gene Products

Several techniques exist that have the ability to detect novel fusion gene products, which arise as a result of chromosomal translocation, inversion, or deletion. Some examples are listed in Table 4. These methods do not rely on genomic DNA and are instead RNA-based assays that enable transcriptomic analysis. Therefore, the direct detection of chromosomal structural variation is not possible using these methods. For the examples listed in Table 4, any structural variation that does not result in a fusion gene product is not detected. Similar to most sequencing approaches, these do not provide data on a cell-by-cell basis and require researchers to choose a specific gene or region to focus on.

Basic to the vast majority of genome editing strategies is the requirement for intentional induction of targeted DNA DSBs; thus, some degree of DNA mis-repair/mis-rejoining is unavoidable and to be expected. The combination of endogenous DSBs, off-target DSBs, and those introduced by the gene editing itself makes it highly likely that the products of mis-rejoining will result in a heterogeneous population of cells containing a variety of low-frequency structural variants. Although sequencing is widely used as a metric for measuring mis-repair events, numerous recent studies have shown that sequencing-based approaches to date are not sufficient, nor are they a highly reliable means for quantitative assessment of mis-rejoining events that result in structural rearrangements [17,53,55]. Classical unbiased cytogenetic techniques, such as G-banding, are extremely low resolution and thus unsuitable. Classical targeted cytogenetic techniques, such as break-apart FISH assays, are limited to the detection of a small number of large variants and so are also unsuitable. Modern directional genomic hybridization or dGH—a direct, single-cell measurement of genomic integrity—can efficiently detect heterogenous products of mis-repair at the edit site, between the edit site and off-target sites, and at random locations throughout the genome. For future applications, a unified approach utilizing both state-of-the-art sequencing and dGH-based cell-by-cell monitoring and validation of genome-wide structural variants will provide a more comprehensive understanding of the level of genotoxic events in edited cell populations.

It is important to appreciate that errors arising from the mis-rejoining of DSBs can result in chromosomal rearrangements regardless of the origin of such DNA damage. This problem is amplified when multiple edit sites (DSBs) are introduced concurrently, which increases the probability of mis-rejoining broken DNA ends (Figure 6). The resulting structural variants have the potential to disrupt gene regulation and contribute to disease [87,93]. For example, in a study of aromatase excess syndrome patients, four distinct inversions reversed the transcriptional orientation of their associated promoters, resulting in overexpression of the gene controlling aromatase (*CYP19*) in the seven patients studied [94]. In the context of cancer, approximately 5% of all non-small-cell lung cancer patients have an *ALK-EML4* translocation, which equates to about 70,000 patients per year [95]. Small inversions can contribute to other cancers, including myeloid leukemia, in which an inversion on chromosome 16 results in a *CBFB-MYH11* fusion gene and the subsequent development of disease in 7.6% of patients [96,97]. An inversion on chromosome 10 resulting from improper recombination of *RET* and *H4* genes is associated with papillary thyroid cancer; although these genes are separated by a great linear distance, their proximity in the nucleus allows for this rearrangement to occur [98]. Furthermore, more complex chromosome aberrations and chromothripsis have been associated with many types of cancer via the creation of fusion oncogenes or loss of tumor suppressor genes [31,50,51,99,100]. Clearly, the biological complexity of mis-repair and consequential structural variation can and does lead to alterations in gene expression that contribute to carcinogenesis.

The strand-specificity of the dGH methodology provides a quantitative assay for obtaining genome-wide structure-based information at high-resolution that is not easily obtained or even possible using traditional cytogenetics or sequencing approaches. In a relatively recent study of clones of human fibroblasts isolated after gamma irradiation, samples were processed either by next-generation sequencing (NGS; short-read massively parallel paired-end sequencing) or dGH for the detection of structural abnormalities present on chromosome 3 [17]. In all five clones analyzed, structural variants, including inversions, were readily visualized and identified by dGH. In contrast, success was mixed using the sequencing approach. For one clone, a translocation was identified and the precise location of the breakpoint was identified at the single bp level, but no indication of an inversion was detected despite its clear presence in every cell within the clone by dGH. Even so, we note that technological advances have been and continue to be made with NGS, and further, there are resolution limitations to current dGH assays arising from the physics of fluorescence detection. For instance, definitive locations of breakpoints cannot be identified with dGH assays as typically designed, although these can be used as a reference point to inform subsequent series of targeted dGH experiments to closely approximate breakpoint locations and inform targeted sequencing strategies. Additionally, extremely small variants (<2 Kb) cannot be resolved with the current limit of detection of dGH assays due to the small number of fluorescence signals hybridized to the DNA target, a limitation that can be overcome using standard strategies for signal amplification, such as side labeling the dGH probes or utilizing multiple end labels. The lower detection limit of dGH is also influenced by the particular fluorescence microscope system used, as well as the density and uniqueness of the target genome; for the dGH assay designs described here, it is defined as ~2 Kb or larger.

Improvements to classic CRISPR-Cas9 editing systems have also been developed that do not require direct DSB formation, including base or prime editing [101,102,103]. Although the risks associated with DSB formation are minimized with these approaches, the potential consequences of single-strand cuts in an editing context are currently unknown. There does exist some potential for multiple single-strand nicks/breaks to be converted to DSBs, albeit at a low frequency [43,104,105]. Nevertheless, dGH remains a valuable assay for monitoring DNA damage/repair and instability in genome-edited populations. Additionally, dGH can be used for proof-of-concept testing and validation of the efficiency, safety, and other advantages of new advancements over classic CRISPR-Cas9-based systems.

FDA requirements for testing of genotoxicity and chromosomal aberrations (OECD TG 473) state that such assays must have the ability to detect clastogenicity (e.g., deletions, insertions, and translocations due to treatment) and heteroploidy (e.g., gain of chromosomes) [106]. Currently available assays that can make such cell-by-cell measurements include traditional cytogenetic approaches, like G-banding and standard FISH. dGH can readily detect these variants, as well as multiple complex variants and potentially more gentoxic small inversions, all at high resolution and all concurrently in a single assay (Figure 4, Figure 5, Figure 6 and Figure 7), making it an ideal candidate for genotoxicity testing, as well as for informing and optimizing personalized medicine strategies based on CRISPR/CAS-9 and any genome editing technology that induces intentional (or unintentional) DSBs.

## Figures and Tables

**Table 1:** Available dGH assays and summary of their utility in various assay formats. Mb = megabase; Kb = kilobase.

Technique	Purpose	Details
Chromatid paint dGH (SCREEN) 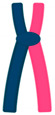	Full coverage of single sister chromatid on target chromosome Can be used to target chromosomes of interest or in a whole-genome format	Discovery of inversions or other aberrations, such as translocations, where location, type, instance, and/or prevalence are unknown Lower limit of detection: >2 KB Detection of off-target effects in edited cell populations
Targeted dGH (In-site) 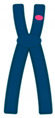	Targeted probe designed for an area of interest	Useful for studying a specific gene/target (i.e., edit site or known disease target) Detection of unintended on-target effects in edited cell populations

**Table 2:** Cytogenetics- and sequencing-based techniques for detection of chromosomal rearrangements.

Method	Purpose	Strengths	Limitations
Giemsa staining/G-banding	Staining of AT-rich regions to create a unique banding pattern for each chromosome Generation of karyograms International standard reference for gene mapping	Identification of large-scale chromosomal aberrations	Low resolution (10 Mb) Cannot detect small inversions or those that do not grossly alter the banding pattern Lack of targeted information
Fluorescence in situ hybridization (FISH)	Fluorescence probe-based detection of target chromosomes. Can be against a specific target region within a chromosome or an entire chromosome (“whole chromosome painting”)	Can detect targeted, specific structures and rearrangements	Lack of commercially available, validated probes for many species and targets Widely varied procedures Cannot directly detect inversions Targeted analysis only
Spectral karyotyping (SKY) or multiplex or multifluor combinatorial FISH (mFISH)	Evolution of FISH that allows for visualization and unique identification of all painted chromosomes in a single hybridization reaction	Simultaneous visualization of all chromosomes Can detect interchange chromosomal rearrangements	Estimated resolution of 0.5–2 Mb for interchromosomal rearrangements [88] Cannot detect inversions or non-lethal deletions
Comparative genomic hybridization (CGH)	Identification of copy number variation	Detection of deletions and amplifications	Cannot detect inversions or translocations
Genomic Vision- Genomic Morse Code (GMC)	High-resolution banding of a genomic region of interest	Detection of structural variants and copy number variants Visualization of hard to sequence regions	Lack of whole-genome information (can visualize up to several Mb) Pooled, double-stranded DNA does not provide data on a cell-by-cell basis. No inversion detection
BioNano-Genome Mapping	Genome mapping using a sequence of probes to generate sequence-based fluorescent patterns	Detection of variants, insertions, and deletions	Pooled, double-stranded DNA does not provide data on a cell-by-cell basis. No inversion detection

**Table 3:** Sequencing-based techniques for the detection of structural variants.

Method	Strengths	Limitations
Targeted sequencing	Specific data about a genomic region of interest	Lack of whole-genome information
Paired-end sequencing	Detection of structural variants based on end pairs that are mapped abnormally far apart on the normal genome sequence	Cannot reliably detect variants in repetitive regions; heterozygous/rare variants
Long-read sequencing	Longer reads allow for improved detection of structural variants in repetitive regions	Cannot reliably detect heterozygous/rare variants High error rate
Single-molecule real-time sequencing (SMRT)	Single DNA template is sequenced with a single DNA polymerase Evolution of long-read sequencing	Cannot reliably detect rare variants due to pooled cell samples Lower throughput due to high cost and time requirements
Unidirectional sequencing	Targeted primer used with bridge adaptors on sheared DNA Often designed for detection of off-target DSBs associated with CRISPR/Cas9	Requires specialized equipment for the shearing of DNA Cannot reliably detect inversions
Single-cell sequencing	Isolation and lysis of single-cell preparations allow for analysis on a cell-by-cell basis rather than population-based information	High level of noise can confound results Low capture efficiency during single-cell isolation Low throughput
10X Genomics Next-GEM sequencing	Combines the specificity of short-read sequencing with the broad range of information captured by long-read sequencing Can detect large structural variants and provide sequence-specific information for single cells	Bioinformatic calculation of structure rather than direct detection makes structural variant detection difficult Susceptible to selection bias

**Table 4:** Techniques for the detection of fusion gene products.

Method	Purpose	Strengths	Limitations
NanoString nCounter	Multiplexed gene expression and biomarker analysis platform that uses fluorescent barcodes for identification of sequences	Multiplexing of regions for analysis of copy number variants Fusion gene detection	Targeted approach; lack of whole-genome information (up to 800 targets) Does not provide data on a cell-by-cell basis
Anchored Multiplex PCR (ArcherDX)	NGS-based targeted approach for detection of genetic variants	Discovery of fusion gene partners Detection of splice variants	Targeted NGS approach (lack of whole-genome off-target effects) Does not provide data on a cell-by-cell basis

## Data Availability

Data are contained within the article.
